# gammaMAXT: a fast multiple-testing correction algorithm

**DOI:** 10.1186/s13040-015-0069-x

**Published:** 2015-11-20

**Authors:** François Van Lishout, Francesco Gadaleta, Jason H. Moore, Louis Wehenkel, Kristel Van Steen

**Affiliations:** 1 Systems and Modeling Unit, Montefiore Institute, University of Liège, Allée de la découverte 10, Liège, 4000 Belgium; 2 Bioinformatics and Modeling, GIGA-R, Avenue de l’Hôpital 1, Sart-Tilman, 4000 Belgium; 3 Institute for Biomedical Informatics, Perelman School of Medicine, University of Pennsylvania, Philadelphia, 19104-6021 PA USA

**Keywords:** Multiple testing, Genome-wide interaction studies, MaxT, Gamma distribution, SNP-environment interactions, 3-order interactions, Algorithmic

## Abstract

**Background:**

The purpose of the MaxT algorithm is to provide a significance test algorithm that controls the family-wise error rate (FWER) during simultaneous hypothesis testing. However, the requirements in terms of computing time and memory of this procedure are proportional to the number of investigated hypotheses. The memory issue has been solved in 2013 by Van Lishout’s implementation of MaxT, which makes the memory usage independent from the size of the dataset. This algorithm is implemented in *MBMDR-3.0.3*, a software that is able to identify genetic interactions, for a variety of SNP-SNP based epistasis models effectively. On the other hand, that implementation turned out to be less suitable for genome-wide interaction analysis studies, due to the prohibitive computational burden.

**Results:**

In this work we introduce gammaMAXT, a novel implementation of the maxT algorithm for multiple testing correction. The algorithm was implemented in software *MBMDR-4.2.2*, as part of the MB-MDR framework to screen for SNP-SNP, SNP-environment or SNP-SNP-environment interactions at a genome-wide level. We show that, in the absence of interaction effects, test-statistics produced by the MB-MDR methodology follow a mixture distribution with a point mass at zero and a shifted gamma distribution for the top 10 % of the strictly positive values. We show that the gammaMAXT algorithm has a power comparable to MaxT and maintains FWER, but requires less computational resources and time. We analyze a dataset composed of 10^6^ SNPs and 1000 individuals within one day on a 256-core computer cluster. The same analysis would take about 10^4^ times longer with *MBMDR-3.0.3*.

**Conclusions:**

These results are promising for future GWAIs. However, the proposed gammaMAXT algorithm offers a general significance assessment and multiple testing approach, applicable to any context that requires performing hundreds of thousands of tests. It offers new perspectives for fast and efficient permutation-based significance assessment in large-scale (integrated) omics studies.

**Electronic supplementary material:**

The online version of this article (doi:10.1186/s13040-015-0069-x) contains supplementary material, which is available to authorized users.

## Background

Personalized medicine proposes to customize healthcare using molecular analysis [1–5]. However, for most human complex diseases, a deeper comprehension of the underlying biology is needed to make this approach workable. Since individual genes usually do not account for much of the heritability of phenotypes, the focus should be on the combined effect of all the genes in the background, rather than on the disease genes in the foreground [6–9]. *MBMDR-4.2.2* is a software dedicated to genome-wide association interaction studies (GWAIs), the purpose of which is to identify pairs of SNPs and/or environmental factors that might regulate the susceptibility to the disease under investigation. The difficulty is to find a good balance between four main issues, that we summarise in the following objectives:

1. Minimize the amount of false discoveries.
2. Achieve sufficient statistical power to detect relevant pairs.
3. Reduce the computational burden implied by the high number of tests for interactions.
4. Provide a versatile software package that accommodates different study designs and study features, including flexibility in trait measurement types and the possibility to adjust for important predictor variables and confounders.

### Available software

Among the numerous software designed for pair-wise or higher-order SNP-SNP interactions, we recall BOOST [10], BiForce [11], epiGPU [12], EpiBlaster [13], GLIDE [14], Multifactor Dimensionality Reduction (MDR) [15, 16] and Model-Based Multifactor Dimensionality Reduction (MB-MDR) [17, 18]. The following comparison of these approaches is mainly inspired from [19] who review and discuss several practical aspects GWAIs typically involve. BOOST is a software based on fast Boolean operations, to quickly search for epistasis associated with a binary outcome. Its main drawbacks are its inability to accommodate missing data and its necessity to perform a multiple testing correction outside the software package. BiForce is a regression-based tool handling binary and continuous outcomes, that can take account of missing genotypes and has a built-in multiple testing correction algorithm. Although, the latter is based on a fast Bonferroni correction implementation, it leads to reduced power for GWAIs, as further discussed in Multiple-testing correction Section. EpiBlaster, epiGPU and GLIDE are all GPU-based approaches. An obvious drawback of GPU-dependent software is that it is tuned for a particular GPU-infrastructure. Therefore, users are advocated to acquire the exact same infrastructure and only experts can adapt the code to specific needs. Note that users willing to work on dedicated hardware to speed up the computations can even turn to field-programmable gate array (FPGa) [20]. MDR is a non-parametric alternative to traditional regression-based methods that converts two or more variables into a single lower-dimensional attribute. The end goal is to identify a representation that facilitates the detection of non-linear or non-additive interactions. Over-fitting issues in MDR are solved via cross-validation and permutations. Since the design of MDR, several adaptations have been made [21]. MB-MDR breaks with the tradition of cross-validation and invests computing time in permutation-based multiple multilocus significance assessments and the implementation of the most appropriate association test for the data at hand. It is able to correct for important main effects. Its main asset compared to the other methods is its versatility. MB-MDR can for instance be used to highlight gene-environment or gene-gene-environment interactions in relation to a trait of interest, while efficiently controlling type I error rates. The trait can either be expressed on a binary or continuous scale, or as a censored trait. *MDMDR-3.0.3* is a C++ software tool based on the MB-MDR methodology, achieving good results regarding objectives (1), (2) and (4) [22, 23]. However, concerns about computational efficiency remain when scaling up to exhaustive genome-wide interaction contexts. In this work we introduce a new version of the software, *MDMDR-4.2.2*, based on a novel multiple-testing correction algorithm, with the purpose of improving the performances along objective (3), with the same benefits as before regarding the other three ones.

### Multiple-testing correction

In GWAIs, the most global null hypothesis is that none of the SNPs pairs, nor their main effects, are associated with the trait. Testing each pair independently at level *α* does not control the overall FWER at level *α*; an adjustment is needed for the fact that multiple tests are performed. One such adjustment can be realized via a Bonferroni correction [24]. This is a so called single-step procedure for strong FWER control. Single-step methods tend to be conservative though and improvements in power can be achieved by so called step-down procedures [25]. Among these we recall step down minP adjusted *p*-values (minP) and step down maxT adjusted *p*-values (maxT). These methods guarantee strong control of the FWER under the subset pivotality assumption and weak control under all conditions [26]. Both procedures are available in *MDMDR-3.0.3*, the adjusted *p*-values being estimated by permutation. Since a high number of pairs of SNPs are tested, minP tends to be more conservative than maxT [25]. Furthermore, minP requires more computations than maxT. For these reasons, maxT is the default choice in *MDMDR-3.0.3*. Note that the drawback of maxT compared to minP, is that when the test statistics are not identically distributed unbalanced adjustments can be observed because not all tests contribute equally to the computed adjusted *p*-values.

**Fig. 1:**
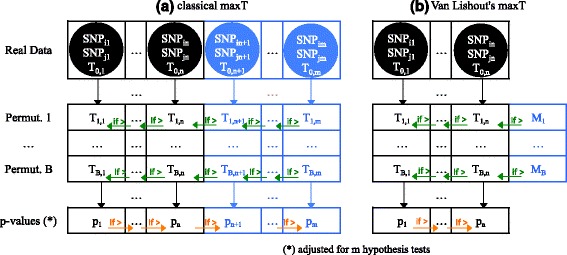
classical MaxT versus Van Lishout’s implementation of MaxT. In the classical implementation of MaxT, all *T*_*i*,*j*_ values are computed and stored in memory, $\forall i=0\dots B$, ∀*j*=1…*m*. Then, *T*_*i*,*j*_ is overwritten by *T*_*i*,*j*+1_ whenever *T*_*i*,*j*+1_>*T*_*i*,*j*_, $\forall i=1\dots B$, ∀*j*=*m*−1…1. Finally, *p*_*j*+1_ is overwritten by *p*_*j*_ whenever *p*_*j*_>*p*_*j*_+1, ∀*j*=1…*m*−1. In Van Lishout’s implementation of MaxT, the [*T*_*i*,*n*+1_,…,*T*_*i*,*m*_] values are computed as before $\forall i=1\dots B$, but only the maximum values *M*_*i*_ are stored in memory (for *i*>0)

Figure 1(a) describes the classical implementation of maxT in MB-MDR. Test-statistics are computed for all *m* pairs of SNPs and sorted in decreasing order in vector *Real Data*. The trait is permuted *B* times and test-statistics are computed for all pairs of SNPs and stored in vectors *Permutation*_*i*_, *i*=1,…,*B*. The latter are browsed from right to left and any value higher than its left neighbor’s value overwrites the latter value. This step is an algorithmic trick to reach efficiently an idea that is best explained the other way around. Let *T*_*i*,*max*_ be the maximum of *Permutation*_*i*_, *i*=1,…,*B*. The *T*_*i*,*max*_ values can be used to approximate the distribution of the highest observed value when testing *m* pairs under the global null hypothesis (no pair of SNPs associated to the disease). Comparing *T*_0,1_ to this distribution enables the computation of adjusted *p*-value *p*_1_, i.e. the probability of observing a value as extreme as *T*_0,1_ for the most promising pair of SNPs. Removing the latter from the data and restarting the whole procedure would obviously allow the computation of adjusted *p*-value *p*_2_ and so on for the remaining ones. From an algorithmic point of view, this would be a waste of time, hence the aforementioned procedure leading to the same result. Finally, the adjusted *p*-values are browsed from left to right and any value higher than its right neighbors’s value overwrites the latter. This procedure obviously aims at controlling the FWER. A particular hypothesis can indeed now only be rejected if all hypotheses were rejected beforehand. The problem of the original maxT is that it is both time and memory consuming.

Van Lishout’s implementation of maxT solves the latter issue [23]. It is based on the observation that in practice, only a few adjusted *p*-values will point towards interesting interactions to investigate. With this in mind, it adapts the original method such that it still calculates the test-statistics of all pairs, but only computes the adjusted *p*-values of the *n* best pairs, i.e. the ones with the *n* lowest adjusted *p*-values. The default value is *n*=1000 and can be tuned without loss of generality according to the researcher’s needs. Note that despite the fact that only *n* adjusted *p*-values are produced, they are still adjusted at the overall level, i.e. for the *m* association tests. Figure 1(b) describes Van Lishout’s MaxT implementation. The different steps are reported in Table 1.

**Table 1:** Van Lishout’s MaxT

(1) Compute the test-statistics for all pairs, but only store the *n* highest tests values. The result is a *Real data*
vector where *T*_0,1_≥*T*_0,2_≥…≥*T*_0,*n*_.
(2) Initialise a vector *p* of size *n* with 1’s.
(3) Perform the following operations for *i*=1,…,*B*:
(a) Generate a random permutation of the trait column.
(b) Compute *T*_*i*,1_,…,*T*_*i*,*n*_ and store them in a *Permutation*_*i*_ vector.
(c) Compute the maximum *M*_*i*_ of the test-statistics values *T*_*i*,*n*+1_,…,*T*_*i*,*m*_.
(d) Replace *T*_*i*,*n*_ by *M*_*i*_ if *T*_*i*,*n*_<*M*_*i*_.
(e) Force the monotonicity of the *Permutation*_*i*_ vector: for *j*=*n*−1,…,1 replace *T*_*i*,*j*_ by *T*_*i*,*j*+1_ if *T*_*i*,*j*_<*T*_*i*,*j*+1_.
(f) For each *j*=1,…,*n*, if *T*_*i*,*j*_≥*T*_0,*j*_ increment *p*_*j*_ by one.
(4) Divide all values of vector *p* by *B*+1 to obtain the *p-values* vector. Force monotonicity: for *j*=1,…,*n*−1,
replace *p*_*j*+1_ by *p*_*j*_ if *p*_*j*+1_<*p*_*j*_.

### Bottlenecks of Van Lishout’s maxT

Van Lishout’s implementation of maxT still leaves room for improvement. In what follows, we identify its main bottlenecks, in order to improve the overall performance on large-scale data. In Table 2 we report the number of operations performed (with the default parameters of the software *n*=1000 and *B*=999) on a dataset containing 10^6^ SNPs, which is equivalent to *m*≈5×10^11^ pairs of SNPs.

**Table 2:** Analysis of the computing times of the different steps of Van Lishout’s implementation of MaxT on a dataset containing 1 million SNPs

	Theoretical value	Numerical value
Step 1	*O*(*m*)	*O*(10^11^)
Step 2	*O*(*n*)	*O*(10^3^)
Step 3 (a)	*O*(*B*)	*O*(10^3^)
Step 3 (b)	*O*(*B* *n*)	*O*(10^6^)
Step 3 (c)	*O*(*B* *m*)	*O*(10^14^)
Step 3 (d)	*O*(*B*)	*O*(10^3^)
Step 3 (e)	*O*(*B* *n*)	*O*(10^6^)
Step 3 (f)	*O*(*B* *n*)	*O*(10^6^)
Step 4	*O*(*n*)	*O*(10^3^)

Table 2 reflects that in step 1 of Van Lishout’s maxT, as many elementary computations are carried out as there are SNP pairs to test. Although significance assessment can be based on fewer SNP pairs, this first step of computing test values and ordering them cannot be avoided nor simplified. However, the most computationally intensive part of the significance assessment procedure is step 3(c). With 10^6^ inputted SNPs, the number of elementary computations required is proportional to 10^14^. Therefore, any improvement at this stage will lead to better overall performances. In “Methods” section, we introduce a novel algorithm for multiple testing, based on Van Lishout’s implementation of maxT. It is implemented in the software *MBMDR-4.2.2* and resolves remaining concerns about maxT’s computation time in genome-wide screens for genetic interactions using the MB-MDR framework.

## Methods

In *MBMDR-4.2.2* the value of *M*_*i*_ from Fig. 1 will be estimated from a sample from [ *T*_*i*,*n*+1_,…,*T*_*i*,*m*_] rather than calculated exactly. A detailed explanation of how we perform such an improvement is provided in the next section.

### Distribution of MB-MDR statistics

We have indicated before that MB-MDR offers a flexible framework to test for SNP-SNP interactions. The software in which the framework is implemented has a modular built-up that allows a flexible choice of association test, depending on the input data. For instance, for quantitative traits, t-tests or non-parametric equivalents can be carried out. For binary traits, chi-squared test of independence can be chosen. The association test that best reflects the data at hand is used in both stage 1 and stage 2 of the MB-MDR framework [27]. After the data manipulation of combining cells using trait information, MB-MDR’s final test statistic no longer follows the theoretical sample distribution of the initially chosen test statistic. In fact, earlier work has shown that such sequential pooling may lead to permutation-based distributions of within MB-MDR test statistics that depend on the number of multi-locus genotype cells pooled [28] or on the minor allele frequencies (MAFs) of the SNP pair under consideration [29]. Rather than looking at the null distribution of the test statistic linked to a SNP-pair, we are now interested in the distribution of a number of test values over several SNP-pairs, from which to derive the maximum value *M*_*i*_. We hypothesize that test values in [ *T*_*i*,*n*+1_,…,*T*_*i*,*m*_], with *i*>0, follow a mixture distribution of a shifted gamma distribution [30] and a point mass at zero. Note that zero test values are induced by scenarios for which the MB-MDR test statistic cannot be computed. In *MBMDR-4.2.2*, whenever a group of subjects (e.g., in a 2-SNP interaction study, those subjects having two copies of the minor allele at each locus) is compared to the remaining subjects with respect to the trait under study and by using an appropriate association test statistic, this group can either be associated to a higher “risk” (“H” category), a lower “risk” (“L” category) or undecisive “risk” (nor “H”, nor “L”; “O” category) for the trait. Here, “risk” is used loosely. For instance for continuous traits, the “risk” categories above may rather refer to increased (“H” category), decreased (“L” category) mean trait values. Also, in the MB-MDR methodology, risk scales can be refined to incorporate multiple risk categories. It is important to realize that if all subjects are assigned the same label (in this scenario, most probably the “O” label), then MB-MDR will return an exact zero. It is not surprising that lack of power of GWAIs (which depends on sample size but also true effect size) will induce such technical zeros for a significant proportion of the tested SNP pairs. In order to take this important amount of zeros into account, we use the approach described in [31]. We assign a discrete probability mass to the exact zero value. Hence, if ${\mathcal {X}}_{i}$ is a random variable returning a random value from [ *T*_*i*,*n*+1_,…,*T*_*i*,*m*_], with *i*>0, we can define the probabilities $\pi = P({{\mathcal {X}}_{i}} > 0)$ and $1-\pi = P({{\mathcal {X}}_{i}} = 0)$. Therefore, the distribution of ${\mathcal {X}}_{i}$ is semi-continuous with a discontinuity at zero. This implies that the probability density function is $$ {f}_{{\mathcal{X}}_i}(x)=\left(1-\pi \right)\delta (x)+\pi {g}_{{\mathcal{X}}_i}(x){1}_{\left(x>0\right)} $$, where *δ*(*x*) is a point probability mass at *x*=0, $g_{{\mathcal {X}}_{i}}(x)$ is the distribution of the strictly positive values and $\mathbbm {1}_{(x>0)}$ is an indicator function taking the value 1 if *x*>0 and 0 otherwise. The parameter *π* depends on the data at hand and can be estimated with the Maximum Likelihood Estimation (MLE) method [32] from the observed frequency in a sample from [ *T*_*i*,*n*+1_,…,*T*_*i*,*m*_]. Due to the fact that our main goal consists in predicting a maximum, we are not particularly interested in fitting the distribution of $g_{{\mathcal {X}}_{i}}(x)$ on the entire set of strictly positive values. As a matter of fact, fitting the tail of $g_{{\mathcal {X}}_{i}}(x)$ should suffice. We show in the next section that focusing on the top 10 % strictly positive values is an acceptable practical choice. We consider this a good tradeoff between fitting on a large and a smaller range of positive values. The former might lead to a poor fit of the tail, because many samples might not belong to that range. The latter might lead to a poor fit of the tail due to an insufficient number of samples. The amount of values belonging to the top 10 % strictly positive values in [ *T*_*i*,*n*+1_,…,*T*_*i*,*m*_] is given by $q=\frac {(m-n)\pi }{10}$.

#### Assumption 1

We assume that the shifted gamma distribution is a good fit to the tail of $g_{{\mathcal {X}}_{i}}(x)$. Hence, if ${\mathcal {Y}}_{i}$ is a random variable returning a value from the aforementioned top 10 % of strictly positive values, we postulate that its cumulative distribution function (CDF) is given by $F_{{\mathcal {Y}}_{i}}(\,y) = \frac {\gamma \left (k,\frac {y-y_{0}}{\theta }\right)}{\Gamma (k)}$, where *γ* is the lower incomplete gamma function, *y*_0_ is the location parameter, *k* is the shape parameter and *θ* the scale parameter. Some authors discourage the use of the gamma distribution for model fitting due to the difficulty of parameter estimation [33]. However, in the specific case of fitting the tail of the distribution of the MB-MDR statistics, we believe that simpler models would be consistently inaccurate. Moreover, the lack of knowledge regarding the shape of a plausible distribution and the diversity of the data we are performing our computations on, make a versatile distribution function like the gamma, a reasonable assumption. Note that the choice of shifting the gamma distribution comes naturally due to the fact that the smallest strictly positive value should not be in the neighborhood of zero. Indeed, a small value would represent a low-significant association between the interaction of the two loci and the phenotype. As previously mentioned, this would lead to the “O” category for all subjects and an exact zero. The CDF of the random variable ${\mathcal {Z}}_{i}$ returning the maximum of the *q* values belonging to the top 10 % strictly positive values in [ *T*_*i*,*n*+1_,…,*T*_*i*,*m*_] is given by $F_{{\mathcal {Z}}_{i}}(z) = \left [\frac {\gamma \left (k,\frac {z-y_{0}}{\theta }\right)}{\Gamma (k)}\right ]^{q}$. Indeed, if we take *q* independent and identically distributed (i.i.d.) values *y*_1_,*y*_2_,…,*y*_*q*_, then $P[\!(\,y_{1} \le y_{t})\wedge (\,y_{2} \le y_{t}) \wedge \ldots \wedge (\,y_{q} \le y_{t})] = [\!F_{{\mathcal {Y}}_{i}}(y_{t})]^{q} = F_{{\mathcal {Z}}_{i}}(z)$.

#### Assumption 2

We postulate that the parameters *π*, *y*_0_, *k* and *θ* are stable from one permutation to another. This assumption is a plausible one, given the results in Table 3, which show low variance of these parameters across 999 permutations. An analogous observation has been noticed in a similar work [34], based on hypothesis testing with an extreme value distribution. In order to reduce the computational burden of the fitting, we estimate the parameters once every 20 permutations. We consider this a compromise between robustness and performance.

**Table 3:** Mean and variance of the fitted parameters for datasets *D*_1_−*D*_4_

	D _1_	D _1_	D _2_	D _2_	D _3_	D _3_	D _4_	D _4_
	Mean	Var	Mean	Var	Mean	Var	Mean	Var
**π**	0.337	1.247×10^−6^	0.335	3.815×10^−6^	0.137	4.948×10^−7^	0.366	9.356×10^−7^
*y*_0_	7.742	5.566×10^−4^	7.825	8.778×10^−4^	6.189	6.472×10^−4^	7.788	3.805×10^−4^
**k**	1.017	2.612×10^−4^	1.012	2.534×10^−4^	0.990	3.580×10^−4^	1.017	1.725×10^−4^
**θ**	1.917	1.462×10^−3^	1.974	1.532×10^−3^	1.694	1.829×10^−3^	1.917	9.695×10^−4^

### Estimating the parameters of the shifted gamma distribution

As mentioned in the introduction, the gammaMAXT algorithm only differs from Van Lishout’s implementation of maxT (Table 1) with respect to step 3(c). In the novel implementation the maximum *M*_*i*_ is estimated from a sample of size *S*=10^6^ of strictly positives values in [ *T*_*i*,*n*+1_,…,*T*_*i*,*m*_] rather than calculated directly. The parameter *π* is estimated on the fly using a variable *z*, counting the amount of zeros encountered during the sampling process. The new step 3(c) is described in Table 4.

**Table 4:** Step 3(c) of gammaMAXT

(1) If (i modulo 20 = 1) estimate *π*,*y*_0_,*k* and *θ*:
(a) Set *z*=0. Create vector *v* of size S.
(b) Randomly select integer *r* in [*n*+1,*m*].
(c) If *T*_*i*,*r*_=0, z=z+1, else store *T*_*i*,*r*_ in *v*.
(d) Repeat steps (b) and (c) until *v* is full.
(e) Sort *v*. Remove the 90 % lowest values. The new size of *v* is $N=\frac {S}{10}$.
(f) Estimate $\pi = \frac {S}{z + S}$.
(g) Estimate *y*_0_ by the minimum of *v*.
(h) Estimate *k*: see below.
(i) Estimate $\theta = \frac {1}{kN}\sum \limits _{i=1}^{N} (v[i]-y_{0})$.
(2) If (i modulo 20 ≠ 1), use the latest estimated values of *π*,*y*_0_,*k* and *θ*.
(3) Sample *M*_*i*_ from the distribution of the maximum, whose CDF is $F_{{\mathcal {Z}_{i}}}(z) = \left [\frac {\gamma \left (k,\frac {z-y_{0}}{\theta }\right)}{\Gamma (k)}\right ]^{\frac {(m-n)\pi }{10}}$.

Whereas estimates in steps (1)(f), (1)(g) and (1)(i) are obtained via Maximum Likelihood, the estimation of the parameter *k* requires more elaboration. According to [35], an acceptable initial guess being within 1,5 % of the correct value is given by $k = \frac {3-s+\sqrt {(s-3)^{2} + 24s}}{12s}$, with $s = ln \left (\frac {1}{N} \sum \limits _{i=1}^{N} (v[\!i]-y_{0})\right) - \frac {1}{N} \sum \limits _{i=1}^{N} ln(v[i]-y_{0})$. This initial guess is updated iteratively via the Newton-Raphson method [36]. In particular, in every iteration, *k* is updated as $k = k - \frac {ln(k) - \psi (k) - s}{\frac {1}{k} - \psi '(k)}$ until the difference between the new and the old value of *k* is lower than the desired precision (default: 0.000001). *ψ*(*k*) and *ψ* ^′^(*k*) are respectively the digamma and trigamma functions. Finally, Table 5 describes the procedure used at step (3) to compute the final *M*_*i*_ estimation. Note that we have to sample and not take the expectation, in order to mimic the original maxT algorithm.

**Table 5:** Sample *M*_*i*_ when CDF is $F_{{\mathcal {Z}}_{i}}(z)$

(a) Take a too high initial guess of *M*_*i*_ (default: 1000). Initialize variable *b* to half of this value.
(a) Randomly select a real number *r*_*n*_∈[ 0,1].
(c) If $F_{{\mathcal {Z}}_{i}}(M_{i})$ is lower than *r*_*n*_, *M*_*i*_=*M*_*i*_+*b*, else *M*_*i*_=*M*_*i*_−*b*. Divide *b* by 2.
(d) Repeat step (c) until *b* is below the desired precision (default: 0.000001).

### Parallel workflow

**Fig. 2:**
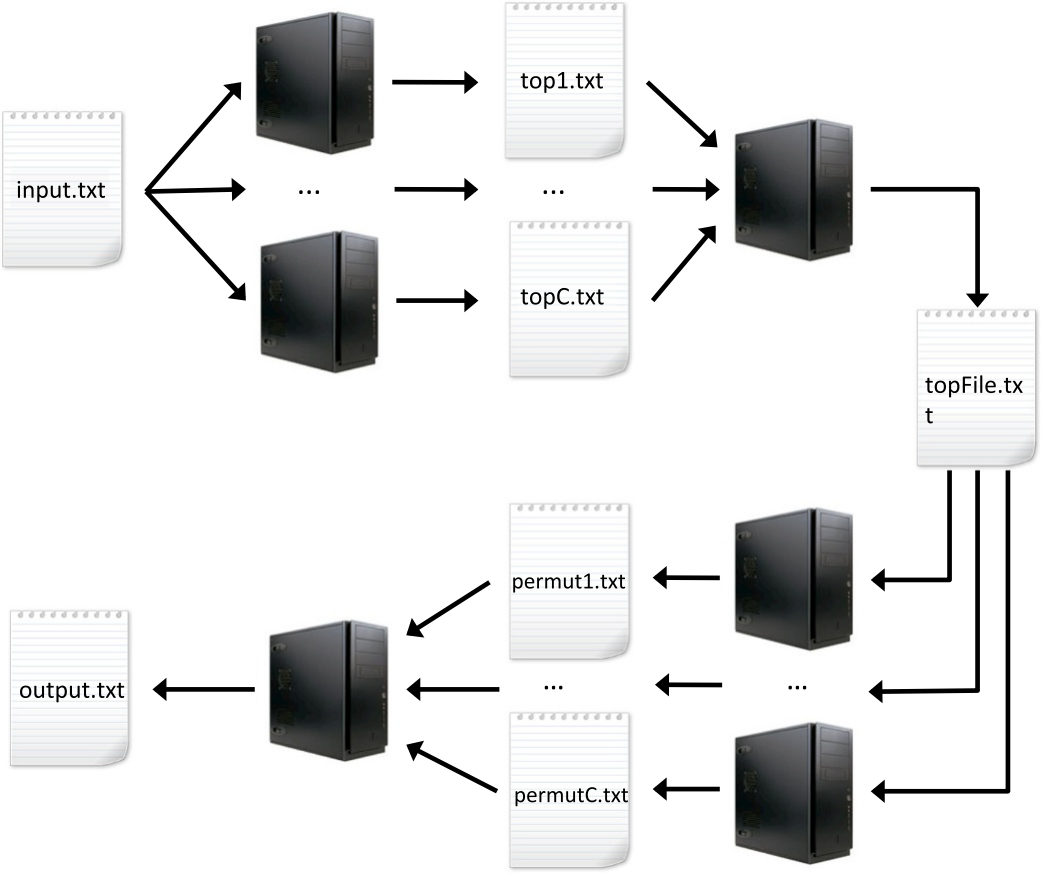
*MBMDR-4.2.2* parallel workflow. First, each cluster node performs a fair proportion of the *T*_0,1_,…,*T*_0,*m*_ values from Fig. 1 and saves the *n* highest into file *top_c.txt*. Second, a node aggregates all *top_c.txt* files and retrieves the overall *n* highest values, saved in *topfile.txt*. Third, each cluster node reads *topfile.txt* and performs an equitable fraction of the *B* permutations of Fig. 1, saving results into file *permut_c.txt*. Finally, a cluster node aggregates all *permut_c.txt* and produces the final output file

Figure 2 describes the four steps of the parallel workflow developed to further make *MBMDR-4.2.2* suitable for GWAIs. The detailed algorithm is given in Table 6.

**Table 6:** gammaMAXT parallel workflow

(1) Each cluster node $c=1\dots C$ performs an equitable fraction of the computations of the *T*_0,1_,…,*T*_0,*m*_
values from Fig. 1. The *n* highest values (and corresponding SNP pair indexes) from each node are saved
into file *top_c.txt*.
(2) Upon termination of all computations at the previous step, a cluster node aggregates all *top_c.txt* files and
retrieves the overall *n* highest values (and corresponding SNP pair indexes). Results are saved into *topfile.txt*.
(3) Each cluster node reads *topfile.txt*, initialize a vector *V* of size *n* with 0’s and performs an equitable fraction
of the *B* permutations of Fig. 1. For each permutation *i* attributed to node *c*:
(a) Generate a random permutation of the trait column.
(b) Compute *T*_*i*,1_,…,*T*_*i*,*n*_ and store them in a *Permutation*_*i*_ vector.
(c) Execute step (3)(c) of the gammaMAXT algorithm to estimate *M*_*i*_.
(d) Replace *T*_*i*,*n*_ by *M*_*i*_ if *T*_*i*,*n*_<*M*_*i*_.
(e) Force the monotonicity of the *Permutation*_*i*_ vector: for *j*=*n*−1,…,1 replace *T*_*i*,*j*_ by *T*_*i*,*j*+1_ if *T*_*i*,*j*_<*T*_*i*,*j*+1_.
(f) For each *j*=1,…,*n*, if *T*_*i*,*j*_≥*T*_0,*j*_ increment *V*_*j*_ by one.
Upon completion of all computations on node *c*, save *V* into file *permut_c.txt*.
(4) A cluster node sums all vectors from the *permut_c.txt* files to obtain a vector *p*. All elements of *p* are
incremented by 1 and divided by *B*+1. The monotonicity is forced: for *j*=1,…,*n*−1, replace *p*_*j*+1_ by *p*_*j*_
if *p*_*j*+1_<*p*_*j*_.

## Results and discussion

In this section, we first show results supporting the two assumptions on which the novel algorithm is based. Then, we analyse the performances in terms of computing-time, power and control of the FWER.

### Results supporting assumption 1

In this part, we investigate the hypothesis that the tail of $g_{{\mathcal {X}}_{i}}(x)$ follows a shifted gamma distribution and that fitting the top 10 % of strictly positive values is an acceptable choice. We use the following datasets for this experiment:

- A simulated dataset *D*_1_ expressed on a binary scale, composed of 1000 SNPs and 1000 individuals. Table 7 states the two-locus penetrance table used to generate it. A balanced number of cases and controls is sampled. The minor allele frequencies of the functional SNPs are fixed at 0.5 and those of the non-functional SNPs are randomly generated from a uniform distribution on [0.05, 0.5]. This corresponds to the first of six purely epistatic models discussed in [15]. Furthermore, any value in the dataset had a 5 % chance to be missing.

**Table 7:** Two-locus penetrance table used to create the simulated datasets *D*_1_, *D*_2_ and *D*_3_

	b/b	b/B	B/B
a/a	0	0.1	0
a/A	0.1	0	0.1
A/A	0	0.1	0
- A simulated dataset *D*_2_, with the same properties as *D*_1_, except that the trait is expressed on a continuous scale.
- A simulated dataset *D*_3_, with the same properties as *D*_1_, except that the MAF’s are on average lower, i.e. the non-functional SNPs were randomly generated from a uniform distribution on [0.05, 0.1].
- A real-life dataset *D*_4_ on Crohn’s disease, for which the trait is expressed on a binary scale [37, 38], reduced to 12471 SNPs and 1687 subjects as in [23].

**Fig. 3:**
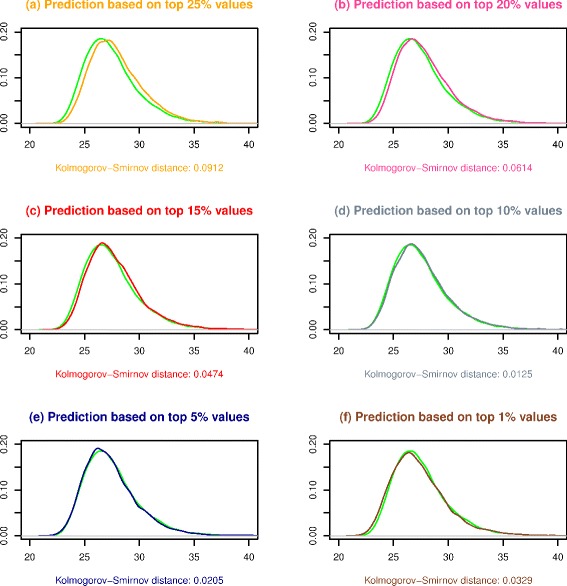
Theoretical (green) versus predicted *M*_*i*_ values for *D*_1_. 10 % is the optimal choice, leading to the lowest Kolmogorov-Smirnov distance

For each of the aforementioned datasets, we first carry out the initial Van Lishout’s implementation of maxT based on 10^4^ permutations to generate a reference distribution for *M*_*i*_. We second execute step (3)(c) of the gammaMAXT algorithm based on 10^4^ permutations, with different values for the internal parameter defining the percentage of strictly positive values belonging to the tail of $g_{{\mathcal {X}}_{i}}(x)$. Figure 3 is generated in R and shows the results for dataset *D*_1_. We observe that focusing on respectively 25, 20, 15, 5 and 1 % of the strictly positive values leads to a good fit, but that 10 % is the optimal alternative. The curves of subfigure (d) are indeed close and the Kolmogorov-Smirnov (KS) distance is the lowest among these choices. This supports the hypothesis that the gammaMAXT algorithm produces accurate predictions of the *M*_*i*_ values. Addditional file 1: Figure S1, Addditional file 2: Figure S2 and Addditional file 3: Figure S3 show that 10 % is consistently a good option, although not always the most optimal one.

### Results supporting assumption 2

In this section, we show results supporting the hypothesis that parameters *π*, *y*_0_, *k* and *θ* are stable across permutations. We perform *MBMDR-4.2.2* analyses on datasets *D*_1_ to *D*_4_, using the default settings. For this experiment, we modified the gammaMAXT algorithm such that it fits new parameters for each of the 999 permutations (not only once every 20 as previously mentioned) and saves these into a file. We report their means and variances in Table 3. We observe that the variance is very low across all scenarios.

### Computing-time of the gammaMAXT algorithm

In order to assess the speed performances of *MBMDR-4.2.2*, we created 4 different datasets with 1000 individuals each, of respectively 10^3^, 10^4^, 10^5^ and 10^6^ SNPs. All datasets were generated using GAMETES, a fast, direct algorithm for generating pure epistatic models with random architectures [39]. Another set of 4 datasets, containing the same number of individuals and SNPs, but expressing the trait on a continuous scale, was created using a similar strategy as for *D*_2_. The parallel workflow of *MBMDR-4.2.2* has been tested on a 256-core computer cluster (Intel L5420 2.5 GHz). The sequential version has been tested on a single core of this cluster. Table 8 shows the results. We observe that *MBMDR-4.2.2* outperforms the computing times of *MBMDR-3.0.3* reported in [23]. For instance, solving a continuous dataset of 10^4^ SNPs on a single core takes about 56 min with *MBMDR-4.2.2* and almost 12 days with *MBMDR-3.0.3*, i.e. about 300 times less. Solving a continuous dataset of 10^6^ SNPs on a 256-core cluster takes about one day with *MBMDR-4.2.2* and would take about 10^4^ longer with *MBMDR-3.0.3*. In general, the theoretical computing time of step 3 (c), which was *O*(*B* *m*) in *MBMDR-3.0.3* according to Table 2, is now independent from *B* and *m*. The computing time of *MBMDR-4.2.2* is therefore asymptotically equal to the computing time of step 1, i.e. *O*(*m*) (a big improvement compared to *O*(*B* *m*), the asymptotic computing time of *MBMDR-3.0.3*). Note that the computing times reported in [23] are based on runs without any correction for the main effects of the SNPS. In this case, the times corresponding to a binary trait are about twice faster than those based on a continuous case. In our study, a codominant correction for the main effects of the SNPs has been performed, implying a regression framework. Since the latter is similar in the binary and continuous case, we logically observe similar computing times.

**Table 8:** Execution times of *MBMDR-4.2.2*. The parallel workflow was tested on a 256-core computer cluster (Intel L5420 2.5 GHz). The sequential executions were performed on a single core of this cluster

SNPs	*MBMDR-4.2.2*	*MBMDR-4.2.2*	*MBMDR-4.2.2*	*MBMDR-4.2.2*
	Binary trait	Binary trait	Continuous trait	Continuous trait
	sequential execution	parallel workflow	sequential execution	parallel workflow
10^3^	13 min 33 sec	20 sec	13 min 18 sec	18 sec
10^4^	52 min 15 sec	1 min 05 sec	56 min 14 sec	53 sec
10^5^	64 h 35 min	22 min 15 sec	70 h 03 min	20 min 28 sec
10^6^	≈ 270 days	25 h 12 min	≈ 290 days	24 h 06 min

The results prefixed by the symbol “ ≈” are extrapolated

### FWER of the gammaMAXT algorithm

To study the control of the FWER, we run *MBMDR-4.2.2* on four sets of datasets:

- A set *S*_1_ of 1000 datasets, each composed of 1000 SNPs and 1000 individuals, containing null data generated randomly from a uniform distribution on [0.05, 0.5]. A balanced number of cases and controls is sampled.
- A set *S*_2_ with the same properties as *S*_1_, except that the trait is expressed on a continuous scale.
- A set *S*_3_ of 200 datasets, each composed of 10^4^ SNPs and 1000 individuals, constructed in the same way as *S*_1_.
- A set *S*_4_ with the same properties as *S*_3_, except that the trait is expressed on a continuous scale.

We report the observed false-positive rates in Table 9. In practice, these are computed as the percentage of datasets containing at least one pair of SNPs that gave rise to an adjusted *p*-value below 5 *%*. On each set, we note that the estimated rates are within the interval [2,5 *%*−7,5 *%*] and satisfies thus Bradley’s liberal criterion of robustness for the significance level *α*=5 *%* [40]. This criterion specifies that the FWER are controlled for any significance level *α*, if the empirical rate $\hat {\alpha }$ is contained in the interval $0.5\alpha \le \hat {\alpha } \le 1.5\alpha $.

**Table 9:** Observed FWER of *MBMDR-4.2.2*

Set	Amount datasets	Observed FWER
*S*_1_	1000	4.5 %
*S*_2_	1000	6.2 %
*S*_3_	200	7 %
*S*_4_	200	6.5 %

### Power of the gammaMAXT algorithm

To evaluate the power, we create nine sets of data with GAMETES. Each set consists in 1000 datasets, all composed of 1000 individuals (500 cases and 500 controls) and 200 SNPs (out of which exactly one pair is linked with the trait). The heritability varies across the datasets from 0.03 to 0.01. In this way, we provide a range of decreasing effect sizes showing the power reduction. Table 10 indicates the percentage of time that the pair linked with the trait gave rise to an adjusted *p*-value below 5 *%*. We observe that the original MaxT and the new gammaMAXT algorithm leads to very similar power. By predicting the *M*_*i*_ values instead of computing them explicitly, we can of course not win power, so that the power of the gammaMAXT algorithm is obviously equal or lower than the one of MaxT. However, we observe that the difference is small, the largest power reduction being of 1,7 %.

**Table 10:** Power comparison between the gammaMAXT and the MaxT algorithms

Heritability	gammaMAXT	MaxT
0.0100	3.7 %	4.2 %
0.0125	17.9 %	19.4 %
0.0150	50.3 %	51.5 %
0.0175	67.0 %	68.7 %
0.0200	86.6 %	87.9 %
0.0225	94.3 %	94.7 %
0.0250	97.5 %	97.8 %
0.0275	99.2 %	99.3 %
0.0300	99.6 %	99.6 %

## Conclusion

In this work we introduced gammaMAXT, a novel implementation of the maxT algorithm for multiple testing correction. The algorithm was implemented in software *MBMDR-4.2.2*, as part of the MB-MDR framework to screen for SNP-SNP, SNP-environment or SNP-SNP-environment interactions at a genome-wide level. In this context, we analyzed a dataset composed of 10^6^ SNPs and 1000 individuals within one day on a 256-core computer cluster. The same analysis would take about 10^4^ times longer with Van Lishout’s implementation of maxT, which was already an improvement of the classic Westfall and Young implementation [26]. These results are promising for future GWAIs. However, the proposed gammaMAXT algorithm offers a general significance assessment and multiple testing approach, applicable to any context that requires performing hundreds of thousands of tests. It offers new perspectives for fast and efficient permutation-based significance assessment in large-scale (integrated) omics studies.

## Availability

*MBMDR-4.2.2* can be downloaded for free at http://www.statgen.ulg.ac.be.

## Additional files

Additional file 1
**Figure S1.** Theoretical (green) versus predicted *M*_*i*_ values for *D*_2_. 10 % is again the optimal choice. (EPS 64 kb)

Additional file 2
**Figure S2.** Theoretical (green) versus predicted *M*_*i*_ values for *D*_3_. 20 % is the optimal choice, but 10 a low Kolmogorov-smirnov distance and remains a good choice. (EPS 64 kb)

Additional file 3
**Figure S3.** Theoretical (green) versus predicted *M*_*i*_ values for *D*_4_. 10 % is again the optimal choice. (EPS 57 kb)
